# Developing a Taxonomy of Communication Techniques and Aids Used By Healthcare Providers During Patient Consultations: Protocol for a Systematic Review

**DOI:** 10.2196/16801

**Published:** 2020-07-14

**Authors:** Violetta Shersher, Terry P Haines, Cylie Williams, Louisa Willoughby, Elizabeth Sturgiss, Carolina Weller

**Affiliations:** 1 School of Primary and Allied Health Monash University Frankston Australia; 2 School of Languages, Literatures, Cultures and Linguistics Monash University Clayton, Victoria Australia; 3 Department of General Practice Monash University Notting Hill, Victoria Australia; 4 School of Nursing and Midwifery Monash University Melbourne, Victoria Australia

**Keywords:** communication, patient-provider communication, patient education, taxonomy

## Abstract

**Background:**

Currently, there is no available standardized taxonomy of defined communication techniques and aids used by healthcare providers during patient consultations. It is challenging to identify communication techniques that contribute to effective healthcare provider and patient consultations and to replicate communication interventions in research.

**Objective:**

The aim of this paper is to describe a protocol for the development and pilot of a taxonomy of communication techniques and aids used by healthcare providers during patient consultations.

**Methods:**

A systematic review will be completed to identify eligible studies. Extracted techniques and aids will be organized into a preliminary taxonomy by a multidisciplinary team. The preliminary taxonomy will be piloted by two groups: research assistants trained in taxonomy application and healthcare providers and healthcare professional students not trained in taxonomy use. The pilot will use custom developed video footage of health provider and patient interactions. Interrater validity and interview feedback will be used to inform a Delphi panel of multidisciplinary healthcare providers and patient experts when they convene to finalize the preliminary taxonomy.

**Results:**

This study was funded in November 2017 by the Monash University Interdisciplinary Research Seed Funding Scheme. Data collection commenced in March 2018, and data analysis is in progress. We expect the results to be published in 2021.

**Conclusions:**

This is the first known attempt to develop a defined and standardized taxonomy of communication techniques and aids used by healthcare providers in patient consultations. The findings will be used to inform future research by providing a detailed taxonomy of healthcare providers’ communication techniques and standardized definitions.

**International Registered Report Identifier (IRRID):**

DERR1-10.2196/16801

## Introduction

Successful communication of information is fundamental to effective therapeutic relationships between healthcare providers [HCP] and patients [1]. However, there is limited understanding of how to measure communication effectiveness during this interaction. An estimated 40%-80% of healthcare information communicated to patients during consultation has been reported to be forgotten immediately, and almost half of the information retained by patients is incorrect [2-4]. Additionally, the amount of information provided by HCPs may be insufficient as patients want more information than they are provided [5]. Further, HCPs have been shown to overestimate the volume of information they provide to patients [6].

HCPs use a range of interpersonal communication techniques, strategies, and aids to convey information to patients in medical consultations. In the healthcare context, the content of interpersonal communication generally falls into the categories of socio-emotional communication, diagnostic communication and problem solving, and the provision of counseling and education [7].

The content of HCP and patient consultations has been classified in The Decision Identification and Classification Taxonomy for Use in Medicine (DICTUM) by Ofstad et al [8]. The DICTUM taxonomy outlines ten categories of physician decisions, comprising of gathering additional information, evaluating test results, defining problem, drug-related, therapeutic, procedure-related, legal and insurance-related, contact related, advice and precaution, treatment goal, and deferment. Each category is defined, and specific actions are outlined. For example, in the category of ‘Physician Statement,’ decision items include ‘Drug refund’ and ‘Sick leave’ [8].

There is no widely accepted complete classification system or taxonomy of the communication techniques used by HCPs to transmit this content. Consequently, there is a common use of terms to describe communication techniques that are not defined clearly and may vary in definitional intent between studies. For example, “attentive listening” or “active listening” is insufficiently defined in the literature, and when a definition is provided, it is often inconsistent [9-16]. The lack of consistent definitions for communication techniques described in the literature creates limitations in the development of interventions, fidelity, study replication, and translation to teaching and practice.

There is a clear need to develop a taxonomy of communication techniques. A taxonomy is a conceptually or empirically derived grouping of objects of interest [17]. Standardized definitions for communication techniques will ensure educational consistency across disciplines and jurisdictions, enable researchers to describe interventions under investigation accurately, identify elements that contribute to overall communication effectiveness, and allow reliable translation of research into clinical practice. Such a taxonomy may be used similarly as the Behaviour Change Technique Taxonomy V1 [18], which has transformed the design and reporting of behavioral change interventions and been cited over 2000 times in the 6 years since its publication.

This protocol describes our method for developing a taxonomy of communication techniques and aids used by HCPs in healthcare consultations with patients.

## Methods

**Figure 1:**
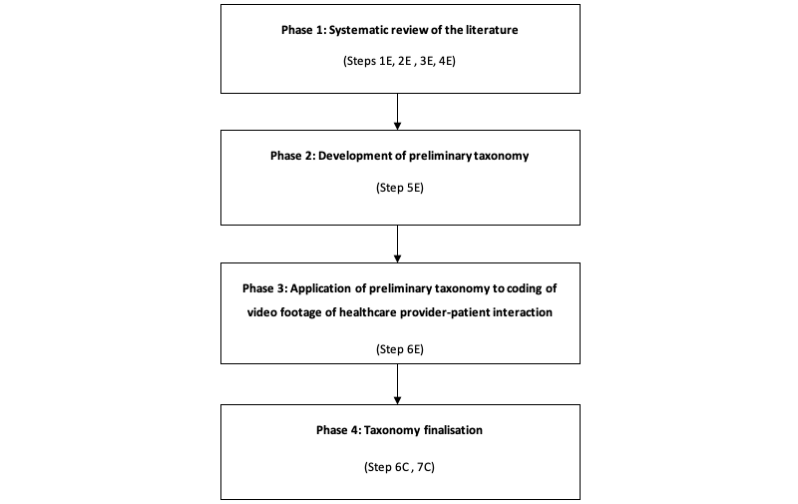
Phases of taxonomy development.

We will adapt the taxonomy development method described by Nickerson et al [19], drawing on the ‘empirical-to-conceptual’ approach for phases one to three. The ‘conceptual-to-empirical’ approach will be used for phase four. Our four-phase process is outlined in Figure 1.

We refer to the equivalent step in Nickerson et al’s seven-step model in parenthesis [19]. The letter ‘E’ indicates the use of the ‘empirical-to-conceptual’ method and ‘C’ indicates the ‘conceptual-to-empirical’ method.

### Phase 1: Systematic Literature Review

In this phase, we identify the range of communication techniques and aids described in the health communication literature and synthesize a definition for each approach.

We developed a set of operational definitions to describe different concepts and measurement units in planning for this phase (Table 1). We consider this to be a necessary step given the diversity and inconsistency in terms used in this field.

**Figure 2:**
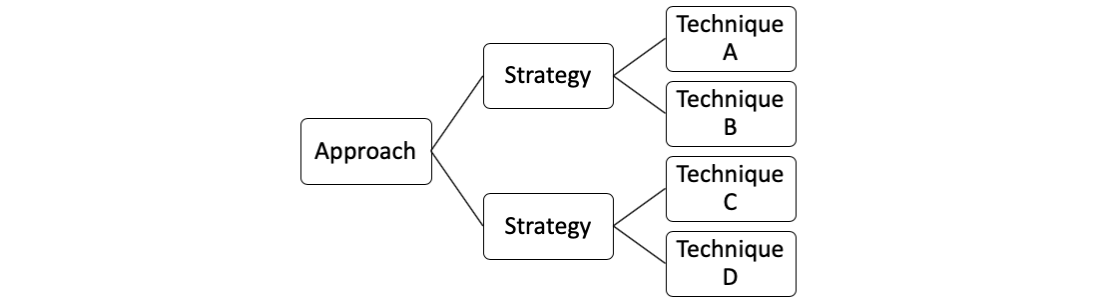
The hierarchical relationship between our operational definitions of communication approaches, strategies, and techniques.

Figure 2 illustrates the hierarchical relationship between our operational definitions of communication approaches, strategies, and techniques.

**Table 1:** Operational definitions.

Term	Operational definition
Patient	Any recipient of health or care services
Healthcare provider	A person who provides preventive, curative, promotional or rehabilitation health care
Caregiver	A family member or paid helper who regularly supports the patient
Communication technique	A single, basic unit of communication method that cannot be broken down into smaller units (eg, nodding)
Communication strategy	A specific combination of communication techniques drawn together to achieve a particular purpose (eg, attentive listening)
Communication approach	A specific combination of communication strategies or models of communication drawn together to achieve a particular purpose or that have a particular effect (eg, patient-centered care)
Communication aid	Visual and audio items that can be used independently of or in conjunction with verbal language to convey information (ie, photos, drawings, illustrations, models, props, graphs, videos or audio recordings)
Written communication aid	Information presented in written form (eg, report, letter, leaflet)

#### Method for Systematic Review

This systematic review will be conducted in accordance with the Preferred Reporting Items for Systematic Reviews and Meta-Analyses (PRISMA) Statement [20]. The protocol has been registered with PROSPERO (Reference/ID No CRD42018095262). We will record our searching and screening process using the bibliographic data management system Covidence.

#### Research Questions

Our systematic review will address the following research question:

What contemporary communication techniques and aids are used by healthcare providers in consultations with patients, as described in the literature?

#### Study Eligibility

The scope of this review will include studies describing interpersonal communication techniques and aids used by HCPs in healthcare consultations with patients.

We will limit the time-span for our search to papers published within the last 10 years to ensure that terms being used to describe techniques being examined are contemporary. Papers providing information only about specific communication approaches (eg, patient-centered care) will be excluded given the volume of hits identified in preliminary searching. Our focus will initially develop the lower levels of the communication technique hierarchy.

The search will exclude several interactions considered out-of-scope for project feasibility: those between HCPs, those involving interpreters or other third parties (eg, family members), and those involving patients who have additional communication needs:

- Third parties (eg, interpreters or family members)
- Doctor-doctor or nurse-nurse interactions
- Profound communication disabilities (eg, aphasia)
- Specialized technological aids (eg, iPads)
- Non-interpersonal health communication (ie, awareness campaigns or radio)
- Communication approaches (eg, patient-centered approach)
- Specialized fields (eg, palliative care)

We anticipate further work may be needed to expand our taxonomy to cover these interactions in the future. The following describes the methods that will be employed in each project phase.

#### Search Strategy for Identification of Relevant Studies

The following terms and Boolean operators will be used when undertaking our search:

“communication style” OR “communication technique*” OR “communication aid” OR “non-verbal communication” OR “verbal communication” OR “communication strateg*” OR “communication repair” OR “communication training” OR “conversation analysis” AND medical OR health OR consultation OR rehabilitation

#### Information Sources

The following databases of published literature will be searched: PubMed, Ovid Medline, CINAHL, Psychinfo, EMBASE, ERIC, Web of Science, and Linguistics and Language Behavior Abstracts.

#### Screening of Abstracts

Two research assistants will independently screen all titles and abstracts for eligibility against the inclusion criteria. Specifically, titles and abstracts will be included if they contain mention of communication techniques or aids used by HCPs in healthcare consultations with patients. Next, the research assistants will independently screen papers at the full-text level for the inclusion criteria. Exclusion reasons will be noted.

Data from eligible studies will be extracted independently by two reviewers using a predesigned data extraction form. The initial 10% of studies will be extracted by both reviewers to identify discrepancies in extraction methods. Where the extracted data differ between assessors, the discrepancy will be resolved by consensus. During the extraction stage, we will categorize the data into broad categories of verbal communication, non-verbal communication, and communication aids.

The data extraction document will contain the following:

- Type of technique/aid: verbal, non-verbal, aid
- Name of communication technique/aid
- Definition of communication technique/aid (if provided)
- Example of communication technique/aid (if provided)
- Additional references: References to an original paper describing a technique or its definition

#### Risk of Bias

We do not plan to undertake an assessment of the risk of bias for this review to the aim is related to the identification of communication techniques, strategies, and aids used by HCPs. We are not seeking to gather information about the effectiveness of outcomes, as this will not add to the development of the taxonomy.

#### Analysis

Descriptive content analysis will be employed, such that each description of a communication technique or aid identified will be coded. Reviewers will then create an operational definition for each code describing a communication technique that seeks to be mutually exclusive from other communication technique codes and describe the smallest “building block” likely to be feasibly useful for describing a communication technique. An exemplar definition of each code will also be added to the operational definition, where one is identified from the reviewed literature. Communication strategies will be coded, but will also be broken down into their subcomponent communication techniques. These techniques, if not already coded, will be added to the pool of communication technique codes.

### Phase 2: Development of Preliminary Taxonomy

In this phase, the aim is to create a thematic structure for taxonomy elements. A multidisciplinary panel will participate in a workshop to develop the prototype taxonomy.

The objectives of the workshop will be to:

1. Collectively review and agree upon definitions of techniques and strategies identified.
2. Allocate specific techniques and aids into categories.
3. Discuss categorizations and reach consensus on taxonomy structure.
4. Identify concerns and discrepancies that will need to be addressed /resolved when finalizing the taxonomy structure in phase 4.

#### Participants

The investigative team will be comprised of professionals from medical, allied health, and applied linguistics fields and patient experts.

#### Procedure

Each communication technique and aid in the data set will be presented to the panel with a label (eg, open-ended question), a synthesized definition, and an example.

These will be presented as individual paper cards. Participants will manually arrange all items into broader categories of type (ie, non-verbal, verbal, aid) and function (eg, information gathering, rapport building, etc). The participants will discuss the allocation of each item and identify discrepancies. The taxonomy structure achieved through discussion and consensus will form the prototype taxonomy.

#### Analysis

An additional research team member independent of those present at the workshop will then review the thematic areas constructed, operational definitions, and exemplars provided for face validity. Suggestions for modification of the structure will be discussed iteratively within the investigative team.

### Phase 3: Application of Preliminary Taxonomy to Coding of Video Footage of Provider-Patient Interaction

We will pilot a practical application of the prototype taxonomy to determine whether the taxonomy and the definitions created can be feasibly and reliably applied to code actual interactions between HCPs and patients. We aim to examine the inter-rater reliability of the application of the taxonomy and the definitions contained within it as a coding framework for recording the use of specific communication techniques used during interactions between HCPs and patients.

We are interested in two contexts:

Context A: The inter-rater reliability likely to be observed if two researchers trained in the application of the taxonomy were seeking to apply the entire taxonomy to recorded communications between HCPs and a patient.

Context B: The inter-rater reliability likely to be observed if HCPs untrained in the use of the taxonomy and students were to apply a restricted section of the taxonomy (while having access to code definitions and examples) to recorded communications between HCPs and patients. This context aims to determine if the operational definitions and examples provided intuitively make sense and give sufficient explanation to allow restricted application without additional training requirements.

#### Participants

Context A: Two members of the investigative team will review the video materials.

Context B: We will recruit 12 health professionals and 12 HCPs students. The health professionals and students untrained in the use of the taxonomy will be drawn from a range of disciplines. The student participants will be enrolled in a health professional degree program at Monash University.

#### Development of Video Footage

We will custom develop simulated interactions between HCPs and patients for video-recording. We will script these videos purposefully such that the maximum frequency across all of the videos of any one communication technique is no higher than 80% and no lower than 20%. Scripting will ensure variability in the frequency of presentation for each particular technique being assessed for coding reliability. Each video will be approximately one minute in length. A total of ten videos will be developed. The taxonomy will be segmented into sections (eg, expressing empathy), and each video segment will demonstrate a set of codes.

#### Procedure

Context A: The videos will be developed by two research team members (TH, VS). The script for each video with notations for when each communication strategy and technique will be employed will serve as the gold standard. Two investigators independent of these research team members who developed the video will be provided with training on the application of the full taxonomy. They will apply coding of the video footage across the full taxonomy. Each code selected will be timestamped. The two investigators coding the video footage will be able to stop, pause, and rewind the video footage as often as needed until they are satisfied that they have completed the coding for the entirety of the taxonomy. The time taken to complete this task for each investigator for each video will be recorded. Investigators coding the video footage will then be interviewed to identify the sections of the taxonomy they found difficult to apply, and sections of the video footage to which they found it more or less difficult to apply the taxonomy.

Context B: The HCP and HCP student participants untrained in the use of the taxonomy will receive a section of codes from the provisional taxonomy. The provisional taxonomy will have been divided into sections based on the categories developed in Phase 2. Four sections of the provisional taxonomy will be purposively selected from the total number for use in this part of the investigation. Each selected section will be provided to a participant using a permuted block randomization, such that 3 students and 3 health professionals are provided with each of the 4 selected sections of the taxonomy. All participants will access the video footage and coding forms on an online portal. They will attempt to apply the code of the video footage across their allocated section of the provisional taxonomy.

Each code selected will be timestamped. Participants will be able to stop, pause, and rewind the video footage as many times as they prefer until they are satisfied that they have completed the coding for their section of the taxonomy. The time taken to complete this task for each participant for each video will be recorded. Participants coding the video footage will be asked open-ended questions to identify the codes they found difficult to apply, and sections of the video footage to which they found it more or less difficult to apply the taxonomy. All participants will complete a debrief task to provide written feedback regarding the feasibility of the taxonomy use and whether the video footage is self-evident.

#### Analysis

Context A: We will examine the agreement between raters by reporting the proportion of communication code items within the prototype taxonomy for which there was agreement within each video, and by reporting Kappa coefficients. For this, each item within the prototype taxonomy will be considered a dichotomous unit of measurement coded as being present within the video or not present. Pairwise comparisons will be made between each investigator rater, and between these raters and the gold standard rating inherent within the script used to develop each video.

Context B: A similar approach will be used for context B; however, a Kappa coefficient for multiple raters will be used. Kappa coefficients will be reported separately for each section of the prototype taxonomy and the health professional vs student HCP participant groups.

The verbal and written feedback obtained from both contexts’ participants will be subject to content analysis with specific items being identified as problematic and reasons behind this being used in the subsequent Delphi panel phase (phase 4) of this research. Additionally, the mean (SD) duration of time taken to complete taxonomy coding for both contexts will be calculated.

### Phase 4: Taxonomy Finalization Workshop

The prototype taxonomy will be revised and finalized by a face-to-face multidisciplinary Delphi panel using the results of the pilot application of the prototype taxonomy to the coding of video scenarios (phase 3). The panel will be comprised of healthcare professionals who are independent of the investigative team, come from a range of disciplines, and patient experts. The panel will be asked to consider the evidence from phases 1, 2, and 3 and consider whether changes to the prototype taxonomy are needed. Each suggested change will then be voted on anonymously by the Delphi panel with reasons for supporting or not supporting the change being written down and read out after the vote by a panel facilitator. Upon hearing these reasons and the results of the first round of voting being read out, participants will be provided with another opportunity to vote on the proposed change and provide reasons why a change was or was not supported. This process will repeat until a consensus is achieved (ie, a change is unanimously endorsed or not endorsed), or a change in the voting is not achieved in two consecutive rounds. In this circumstance, a majority decision will be used to shape the taxonomy; however, a note will be made that the particular change was not universally supported. This process will be repeated for each suggested change until the definitive model of the taxonomy is reached.

## Results

This study was funded in November 2017 by the Monash University Interdisciplinary Research Seed Funding Scheme. Data collection commenced in March 2018, and data analysis is in progress. We expect to publish the results in 2021.

## Discussion

Our systematic review of the literature and developed taxonomy will provide a detailed and defined classification of verbal and non-verbal communication techniques and aids available for HCPs to use with patients in healthcare consultations. We anticipate that this review will be of interest to HCPs, researchers, quality improvement departments at hospitals, clinics, and universities, and policymakers. The findings will be used to inform future research by providing a detailed taxonomy of HCPs’ communication techniques and standardized definitions.

The importance of this work will manifest across a range of areas. First, it will provide a framework and tools for researchers to measure and understand the impact of different communication techniques on patient outcomes. Second, it will provide a framework on which HCP educators may structure their training programs. Third, it will help promote health literacy in patients, which is held as an area where improved communication by HCPs could promote improved health outcomes. Health literacy is defined as the “degree to which individuals have the capacity to obtain, process, and understand basic health information and services needed to make appropriate health decisions” [21]. It has previously been reported by the World Health Organization that low health literacy “significantly drain[s] human and financial resources in the health system” [22]. Future research that directly builds on this taxonomy could investigate the frequency of use of different communication techniques in a range of contexts and for the taxonomy to be adapted to different international settings. Subsequently, additional blue-sky research could identify communication techniques that maximize patient understanding in a range of contexts such as prescribing and health service availability, which may ultimately improve patient health outcomes and minimize adverse outcomes from patient care.

Three study limitations have been identified. First, our search to identify communication techniques and aids is focussed on the published literature. There may be communication techniques being promoted by HCPs and those who train them that have not been described in the published literature, and yet may still be relevant. Second, the team driving this research is based entirely within the Australian context. It is possible that understanding and application of communication techniques applied in this context may be different from that in other countries.

Similarly, differences in cultural norms and patterns of behavior may influence how the investigative team interprets the data arising from literature from other countries and cultures. Also, the Delphi panel recruited for the refinement and finalization stages of the taxonomy will be derived from the Australian context. Future work will be needed to investigate the adaptation of the final model of the taxonomy to other countries and contexts. Finally, the scope of this work excludes communication approaches and techniques used with patients who have profound communication impairments. Again, further work is needed to annex communication approaches, strategies, and techniques to our final model of the taxonomy to support its broader application and benefit.

### Conclusion

To our knowledge, this is the first attempt to create a detailed taxonomy of healthcare consultation communication techniques and aids. The development of this taxonomy can support communication trial design in the future. The availability of this tool will aid researchers to describe better communication techniques and aids used in trials and cohort studies that are founded on communication.

## Abbreviations

HCP: healthcare providers
